# Age-related neurodegeneration and cognitive impairments of NRMT1 knockout mice are preceded by misregulation of RB and abnormal neural stem cell development

**DOI:** 10.1038/s41419-021-04316-0

**Published:** 2021-10-28

**Authors:** James P. Catlin, Leandro N. Marziali, Benjamin Rein, Zhen Yan, M. Laura Feltri, Christine E. Schaner Tooley

**Affiliations:** 1 Department of Biochemistry, Jacobs School of Medicine and Biomedical Sciences, State University of New York at Buffalo, Buffalo, NY 14203 USA; 2 Departments of Biochemistry and Neurology, Jacobs School of Medicine and Biomedical Sciences, Hunter James Kelly Research Institute, State University of New York at Buffalo, Buffalo, NY 14203 USA; 3 Department of Physiology and Biophysics, Jacobs School of Medicine and Biomedical Sciences, State University of New York at Buffalo, Buffalo, NY 14203 USA

**Keywords:** Neurochemistry, Neural stem cells

## Abstract

N-terminal methylation is an important posttranslational modification that regulates protein/DNA interactions and plays a role in many cellular processes, including DNA damage repair, mitosis, and transcriptional regulation. Our generation of a constitutive knockout mouse for the N-terminal methyltransferase NRMT1 demonstrated its loss results in severe developmental abnormalities and premature aging phenotypes. As premature aging is often accompanied by neurodegeneration, we more specifically examined how NRMT1 loss affects neural pathology and cognitive behaviors. Here we find that *Nrmt1*^−/−^ mice exhibit postnatal enlargement of the lateral ventricles, age-dependent striatal and hippocampal neurodegeneration, memory impairments, and hyperactivity. These morphological and behavior abnormalities are preceded by alterations in neural stem cell (NSC) development. Early expansion and differentiation of the quiescent NSC pool in *Nrmt1*^−/−^ mice is followed by its subsequent depletion and many of the resulting neurons remain in the cell cycle and ultimately undergo apoptosis. These cell cycle phenotypes are reminiscent to those seen with loss of the NRMT1 target retinoblastoma protein (RB). Accordingly, we find misregulation of RB phosphorylation and degradation in *Nrmt1*^−/−^ mice, and significant de-repression of RB target genes involved in cell cycle. We also identify novel de-repression of *Noxa*, an RB target gene that promotes apoptosis. These data identify Nα-methylation as a novel regulatory modification of RB transcriptional repression during neurogenesis and indicate that NRMT1 and RB work together to promote NSC quiescence and prevent neuronal apoptosis.

## Introduction

N-terminal methylation of the α-amino group (Nα-methylation) is a highly conserved posttranslational modification that regulates protein/DNA interactions [1–3]. Just over 10 years ago, we identified the first mammalian Nα-methyltransferase, NRMT1 (N-terminal RCC1 methyltransferase 1) [4]. NRMT1 is a highly conserved enzyme that is predicted to Nα-trimethylate over 300 proteins based on a characterized Nα-consensus sequence [4, 5]. Verified targets play roles in mitosis (RCC1), cell cycle (retinoblastoma, RB), DNA damage repair (DDB2), chromatin organization (CENP-B and CENP-A), and transcriptional regulation (SET, MYL9) [3–7]. Accordingly, loss of NRMT1 results in a variety of cellular phenotypes, including aneuploidy, impaired DNA damage repair, and altered cell growth [1, 4, 6, 8]. In breast cancer cells, NRMT1 acts as a tumor suppressor. Its loss increases cell proliferation, cell migration, and xenograft tumor development [8]. Our recent creation of the first NRMT1 knockout (*Nrmt1*^−/−^) mouse has also revealed its importance in development and aging. Approximately 40% of *Nrmt1*^−/−^ mice die within the first month and, of those that survive, only 30% live past 6 months [9]. Those that survive past 6 months exhibit phenotypes associated with premature aging [9].

As premature aging is often accompanied by neurodegeneration [10], we looked more specifically at how NRMT1 loss affects age-related neural pathology and cognitive behaviors. Here we find significant enlargement of the lateral ventricles in *Nrmt1*^−/−^ mice, striatal and hippocampal neurodegeneration, and corresponding behavioral impairments. As the subventricular zone (SVZ) of the lateral ventricles and the subgranular zone (SGZ) of the hippocampus contain the postnatal neural stem cell (NSC) niches [11], we wanted to next determine whether *Nrmt1*^−/−^ mice exhibit abnormal NSC development. We find that after an early expansion, *Nrmt1*^−/−^ mice show premature depletion of the quiescent NSC pool, which is concurrent with an expansion of both the intermediate progenitor cell (IPC) and neuroblast pools, indicating the NSCs are undergoing differentiation. We also see an increase in the number of differentiated neurons remaining in the cell cycle and undergoing apoptosis.

These NSC phenotypes are reminiscent of those seen with loss of the NRMT1 target RB protein [4, 12]. During normal neurogenesis, hypophosphorylated RB inhibits E2F-mediated transcription of cell cycle genes and enables both NSC quiescence and exit from the cell cycle upon terminal differentiation [13]. Phosphorylation of RB during the proliferative phases of neurogenesis releases this inhibition and promotes RB protein degradation and cell cycle entry [14]. Loss of RB results in an expansion of IPCs and neuroblasts within the niches [12, 15]. The resultant IPCs and neuroblasts have time- and context-specific cell cycle and/or differentiation defects, and many ultimately undergo apoptosis [15, 16].

To confirm misregulation of RB in *Nrmt1*^−/−^ mice, we measured its overall protein and phosphorylation levels, as well as expression of its target genes. *Nrmt1*^−/−^ mice exhibit an increase in RB phosphorylation and decrease in overall RB protein, similar to what is seen with RB inactivation [14]. We also see transcriptional de-repression of RB/E2F1 target genes that promote cell cycle entry and identify de-repression of *Noxa*, a target gene that promotes apoptosis [17]. We propose a model where Nα-methylation of RB by NRMT1 promotes its activity as a transcriptional repressor. Loss of NRMT1 promotes RB phosphorylation and release from E2F1, resulting in premature activation of NSC proliferation and neuronal apoptosis. Combined, the depletion of the NSC pool and high rates of neuronal apoptosis would lead to an inability of the niches to replace neurons as they age or become damaged, leading to the observed neurodegenerative and behavioral phenotypes. Taken together, our data identify a new role for NRMT1 in NSC development and suggest its regulation of RB contributes to the maintenance of NSC quiescence and neuronal viability.

## Methods

### Maintenance of *Nrmt1*^−/−^ mice

Homozygous C57BL/6J- *Nrmt1*^−/−^ mice used in the described experiments are generated and genotyped as previously described [9]. Both sexes of mice were used in the molecular studies, their results combined and averaged. The behavioral studies were done with only females, due to the high number of animals required and the need for most *Nrmt1*^−/−^ males to maintain the breeding colony. A priori power analysis was used to predict that eight to ten mice/group will allow for a 20% change between measured variables to be statistically significant in behavioral studies. Mice not needed for experiments were killed by inhalation of carbon dioxide in a clear container followed by cervical dislocation. Mice needed for histological brain sections were killed by perfusion with 4% paraformaldehyde (PFA) following anesthesia. Both the breeding and killing protocols are approved by the SUNY Buffalo Animal Care and Use Committee.

### Barnes maze

The Barnes maze test was performed with minor modifications [18]. Briefly, animals were habituated for 5 min on a circular platform that contained eight equally spaced holes. After habituation, the animals were placed back on the platform for either two or ten training (learning) acquisition trials. During the training phase, an escape hole was present for the animal to enter. A bright light above the platform was used as a weak aversive stimulus to encourage the animal to escape during each training trial. Each animal had up to 3 min to navigate the platform and find the escape hole. Unique shapes and colors on four sides of the maze were used as spatial cues to help the animal locate the escape hole. If the animal was unable to locate the correct escape hole in the 3 min training trial, the animal was gently guided to the correct hole. The learning interval between each animal was 5 min. After two or ten training trials, a test trial was administered 15 min or 24 h after the last training trial, respectively. During the test trial, animals were placed back on the platform for 3 min where correct (T1) and incorrect (T2) hole exploration was measured. Spatial memory index was calculated by: (T1 − T2)/(T1 + T2).

### Open-field and rotarod assays

For open-field assays, animals were placed in a transparent plastic cage (40 × 40 × 30 cm) where they were allowed to freely move around for 30 min. ANYMAZE tracking software (Stoelting, Wood Dale, IL) was used to measure total distance traveled and average speed. Quantification of 5 min bins was used in the analysis. For rotarod assays, animals were placed on an accelerating rotarod (SD Instruments, San Diego, CA) as previously described with minor adaptations [19]. The rotarod machine was programmed to accelerate from 4 revolutions per minute (RPM) to 40 RPMs over a 5 min trial. To prevent injury after falling, thick foam was used to catch the animals. Three training trials were used to familiarize the animal to the test. Ten minutes after the last practice session, two test trials were administered and latency to fall was measured. The average latency of fall between the two test trials was used for analysis.

### QuPath volume analysis

To assess brain volume of specific regions, two sets of slides were created to represent the whole brain (eight sections per set, each spaced 300 μm apart). As a result, the first set of slides contained the rostral half and the second set covered the caudal half of the brain. One full set of cresyl violet stained slides for each animal was scanned using the Aperio ScanScope whole slide scanner (Leica Biosystems, Wetzlar, Germany) with the ×20 objective. Each digitized file was uploaded onto QuPath (version 0.1.2) where they were further processed. Each section containing the brain region of interest was manually traced using the polygon tool. The mouse brain coronal reference of the Allen Brain Atlas was used as a guide for a more accurate trace of the different brain regions. After each trace was completed, an area (μm^2^) given by QuPath was used to calculate the volume of each tissue section. To calculate the volume of a traced region, the formula: area of trace (μm^2^) × thickness of section (30 μm) = volume of traced area (μm^3^) was determined for each section and brain region. To calculate the total volume of a given region, the sum of all volumes calculated for that region were added to give the final volume.

### Immunohistochemistry

Mice were anesthetized and transcardially perfused with 1% phosphate-buffered saline (PBS) followed by 4% PFA before brain removal. Brains were post-fixed in 4% PFA for 72 h and then cryoprotected in a 30% sucrose solution for 1 week. Coronal slices (30 μm) were cut on a Microm HM 525 cryostat (Thermo Fisher Scientific, Waltham, MA) and attached to Superfrost plus slides (Thermo Fisher Scientific). Sections were washed with PBS and blocked in 1× PBS containing 2% Triton X-100 and 5% horse serum for 1 h at room temperature (RT). Sections were incubated overnight at 4 °C with the following primary antibodies in 1 × PBS containing 2% Triton X-100 and 2% horse serum: mouse anti-NeuN (MilliporeSigma, MAB377, Burlington, MA), rat anti-Ki-67 (Thermo Fisher Scientific, 14-5698-82), rabbit anti-Ki-67 (Cell Signaling Technology, 9129S, Danvers, MA), rabbit anti-Sox2 (MilliporeSigma, AB5603), rabbit anti-cleaved caspase-3 (Cell Signaling Technology, 9664S), rabbit anti-Doublecortin (DCX) (Abcam, ab18723, Cambridge, UK), or mouse anti-glial fibrillary acidic protein (GFAP) (Thermo Fisher Scientific, A0237). The following day, sections were washed three times with 1 × PBS and incubated for 1 h at RT in the following secondary antibodies in 1 × PBS containing 2% Triton X-100 and 2% horse serum: Goat anti-rabbit Alexa Fluor 488 or 594 (A11008 or A11012), Goat anti-mouse Alexa Fluor 594 or 647 (A11005 or A2137), or Donkey anti-rat Alexa Fluor 488 or 594 (A21208 or A11007, all Thermo Fisher Scientific). Sections were counterstained with Hoechst 33342 (AnaSpec, Fremont, CA), washed three times in 1× PBS, rinsed in distilled water, and dried at 60 °C for 5 min. Sections prepped for cresyl violet staining were incubated in 0.01% cresyl violet solution containing glacial acetic acid for 5 min. Slides were mounted in vectashield fluorescent anti-fading mounting media (Vector Laboratories, Burlington, Ontario). Slides were visualized using an Aperio ScanScope whole slide scanner (Leica Biosystems), a Cytation 5 Multi-Mode reader (BioTek, Winooksi, VT), a Leica DMi8 fluorescence microscope, or a Leica TCS SP8 spectral confocal. All images captured were at identical regions and exposures between genotypes. Quantification of pixel intensity was performed through ImageJ (NIH) software, where the region or cells of interest were traced and analyzed for mean pixel intensity. Cell counting was also done through ImageJ using the cell counter plugin. Data from medial levels of coronal sections is shown and used in quantifications.

### Quantitative real-time PCR analysis

Lateral ventricles containing the adjacent striatum were collected from 6-week wild-type (WT) and *Nrmt1*^−/−^ mice. Total RNA was extracted using TRIzol reagent (Thermo Fisher Scientific) and converted to cDNA using the SuperScript III first-strand synthesis system (Thermo Fisher Scientific) as previously described [9]. The following primer sets were used: cyclin A2 Fwd 5′-TGAGTTTGATAGATGCTGACCCG-3′, Rev 5′-ATCCAGTCTGTTGTGCCAATGAC-3′; cyclin E2 Fwd 5′-TCTGTGCATTCTAGCATCGACTC-3′, Rev 5′-AAGGCACCATCGTCTACACATTC-3′; *E2f1* Fwd 5′-TGCCAAGAAGTCCAAGAATCA-3′, Rev 5′-CTTCAAGCCGCTTACCAATC-3′; *Apaf1* Fwd 5′-GATGTGGAGGTGATCGTGAAG-3′, Rev 5′-GTAGTGTCGTGGTAGGTCAT-3′; *Puma* Fwd 5′-ATGGCGGACGACCTCAAC-3′, Rev 5′-AGTCCCATGAAGAGATTGTACATGAC-3′; *Noxa* Fwd 5′-AGGAAGGAAGTTCCGCCG-3′, Rev 5′-AGCGTTTCTCTCATCACATCACA-3′, *Hdgf* Fwd 5′-CATGAGAGCCTGTAGCCAC-3′, Rev 5′-GTGGGCTTAGAGGAGAGAG-3′. Reactions were processed in a CFX96 Touch Real-Time PCR System (Bio-Rad, Hercules, CA). Fold change was determined using the ΔΔ CT quantification method. Hepatoma-derived growth factor (*Hdgf*) was used as the housekeeping gene.

### Immunoblotting

Ventricles containing the adjacent striatum were dissected from mice using a brain matrix (Zivic Instruments, Pittsburgh, PA). Tissue was processed with lysis buffer containing Tris (pH 7.6; 15 mM), sucrose (0.25 M), EDTA (2 mM), EGTA (1 mM), sodium orthovanadate (10 mM), sodium fluoride (25 mM), sodium pyrophosphate (10 mM), and the protease inhibitors phenylmethylsulfonyl fluoride, aprotinin, and leupeptin (MilliporeSigma). Lysates were spun at 14,000 RPM for 20 min. Fifteen micrograms of total protein was separated on 10% SDS-polyacrylamide gel electrophoresis gel and transferred to a polyvinylidene difluoride membrane using the Trans-Blot Turbo Transfer System (Bio-Rad). Membranes were blocked using 5% w/v nonfat dry milk in Tris-buffered saline (TBS) with 0.1% Tween 20 (TBS-T) for 1 h at RT. Primary and secondary antibodies were diluted in 5% dry milk/TBS-T solution. Dilutions used for primary antibodies include the following: rabbit anti-RB (1 : 1000; Cell Signaling Technology, 9313S), rabbit anti-phospho RB (Ser795) (1 : 1000; Cell Signaling Technology, 9301T), and rabbit anti-β-Tubulin (1 : 1000; Cell Signaling Technology, 2128L). Secondary antibody dilution was as follows: donkey anti-rabbit (1 : 5000; Jackson ImmunoResearch, 711-035-152). Blots were developed using Clarity Western ECL Substrate (Bio-Rad) on a ChemiDoc imaging system (Bio-Rad).

### Statistics

For the behavioral studies, each experiment was replicated three times with an average of two to three mice from each genotype and results of all trials combined and averaged. The statistical significance of the behavioral studies was analyzed by either two-way analysis of variance or unpaired *t*-test, as indicated in the figure legend. D’Agostino–Pearson and Shapiro–Wilk tests were done to assure normal distribution. No animals were excluded from analysis, no method of randomization was used, and no blinding was done. For molecular analysis, three was used as the minimum number of independent experiments. Three brains for each genotype and age were processed and quantified as described for each experimental procedure; a fourth brain for each genotype was added when samples were available. The statistical significance of the quantifications was analyzed by unpaired *t*-test. Samples were excluded from immunohistochemical analysis due to potential procedural errors as noted by failure of controls to stain. These were replaced by new samples. For all experiments, error bars represent mean ± SEM. *P*-values are included in all figure legends.

## Results

### *Nrmt1*^−/−^ mice exhibit postnatal enlargement of the lateral ventricles and neurodegeneration

**Fig. 1:**
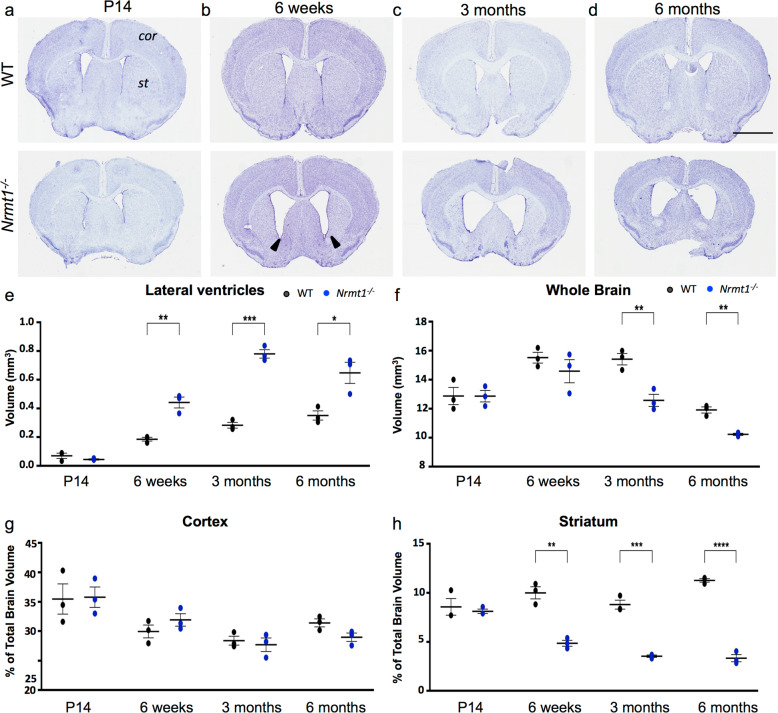
*Nrmt1*^*−/−*^ mice have enlarged lateral ventricles and reduced striatal volume. **a**–**d** Cresyl violet staining showing *Nrmt1*^*−/−*^ mice have enlarged lateral ventricles (black arrow heads) by 6 weeks that persist through 6 months. Quantification of **e** lateral ventricle, **f** whole brain, **g** cortex (*cor*), and **h** striatum (*st*) volume. *p < 0.05, **p < 0.005, ***p < 0.0005, and ****p < 0.0001 as determined by unpaired *t*-test, *n* = 3. Error bars represent mean ± SEM. Scale bar = 2 mm.

*Nrmt1*^−/−^ mice present phenotypes associated with premature aging and phenocopy mouse models deficient in DNA damage repair [9]. As an impaired DNA damage response in neurons has been connected to the onset of age-related neurodegenerative disorders such as Alzheimer’s disease (AD) and amyotrophic lateral sclerosis [20], we sought to determine whether *Nrmt1*^−/−^ mice exhibit any age-related neural pathologies. We first stained whole-brain mounted sections of WT C57BL/6J mice and C57BL/6J-*Nrmt1*^−/−^ mice with cresyl violet to look for gross morphological defects. In 14-day-old postnatal mice (P14), there were no apparent morphological differences between WT and *Nrmt1*^−/−^ brains (Fig. 1a). However, by 6 weeks, *Nrmt1*^−/−^ mice showed a significant enlargement of the lateral ventricles (Fig. 1b, e). This increase became larger at 3 months (Fig. 1c, e) and persisted through 6 months (Fig. 1d, e).

Ventricle enlargement can be due to global changes in brain volume or come at the expense of neighboring regions of gray matter [21]. To determine which is occurring in *Nrmt1*^−/−^ mice, volumes of the striatum, cortex, and total brain were calculated. *Nrmt1*^−/−^ mice did not display global increases in brain volume at any age and overall volume actually decreased with age (Fig. 1f). At 6 weeks, when the ventricle volume increased significantly, overall brain volume did not differ and cortical volume also remained similar (Fig. 1f, g). However, the proportional volume of the striatum to total brain volume was significantly decreased (Fig. 1h), indicating the lateral ventricle enlargement seen in *Nrmt1*^−/−^ mice corresponds to reduced striatal volume. At both 3 and 6 months, *Nrmt1*^−/−^ brains had significantly decreased overall volume (Fig. 1f). The proportional volume of the *Nrmt1*^−/−^ cortex at both these ages showed no difference from WT (Fig. 1g), but the proportional volume of the *Nrmt1*^−/−^ striatum remained significantly different from WT at both 3 and 6 months (Fig. 1h). These data indicate that the lateral ventricle enlargement seen in *Nrmt1*^−/−^ mice occurs concurrently with a decrease in striatal volume.

**Fig. 2:**
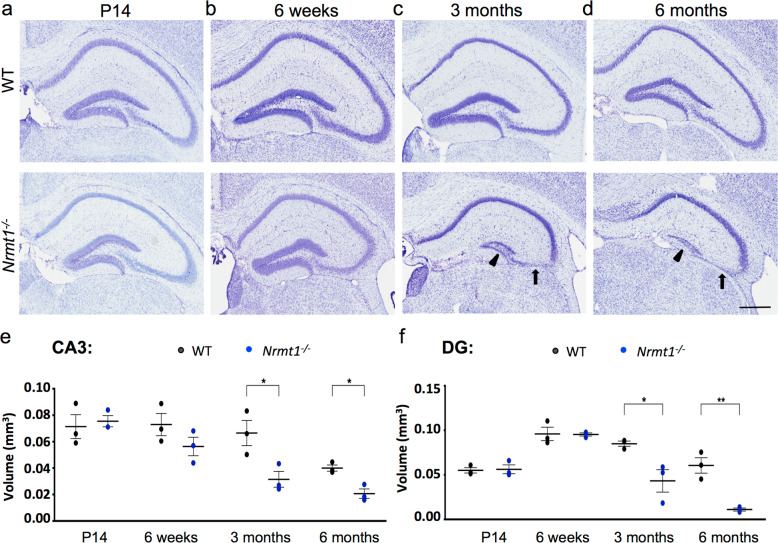
*Nrmt1*^*−/−*^ mice show degeneration of hippocampal neurons. Cresyl violet staining of hippocampal neurons shows similar morphology between *Nrmt1*^−/−^ and WT mice at **a** P14 and **b** 6 weeks. However, at **c** 3 months, *Nrmt1*^−/−^ mice show degeneration of neurons in the CA3 (arrow) and DG (arrowhead). **d** By 6 months, neuronal staining is almost completely absent in the CA3 and DG. Quantification of **e** CA3 and **f** DG volume. *p < 0.05 and **p < 0.005 as determined by unpaired *t*-test, *n* = 3. Error bars represent mean ± SEM. Scale bar = 400 μm.

We next used whole-brain mounted sections to examine the general morphology of the hippocampus at P14, 6 weeks, 3 months, and 6 months. The hippocampal circuit comprises four main regions, the three *Cornu Ammonis* subfields (CA1, CA2, and CA3) and the dentate gyrus (DG). At P14 and 6 weeks, no obvious morphological differences were observed between *Nrmt1*^−/−^ and WT mice (Fig. 2a, b). However, at 3 months, the volumes of both the CA3 and DG are significantly decreased in *Nrmt1*^−/−^ mice, indicating neuronal loss (Fig. 2c, e, f). By 6 months, the volumes of the CA3 and DG are even further decreased, indicating that significant neurodegeneration has occurred by this age (Fig. 2d, e, f). Taken together, these data show that *Nrmt1*^−/−^ mice exhibit both abnormal developmental morphologies and age-related neurodegeneration.

### Behavioral analysis of *Nrmt1*^−/−^ mice

**Fig. 3:**
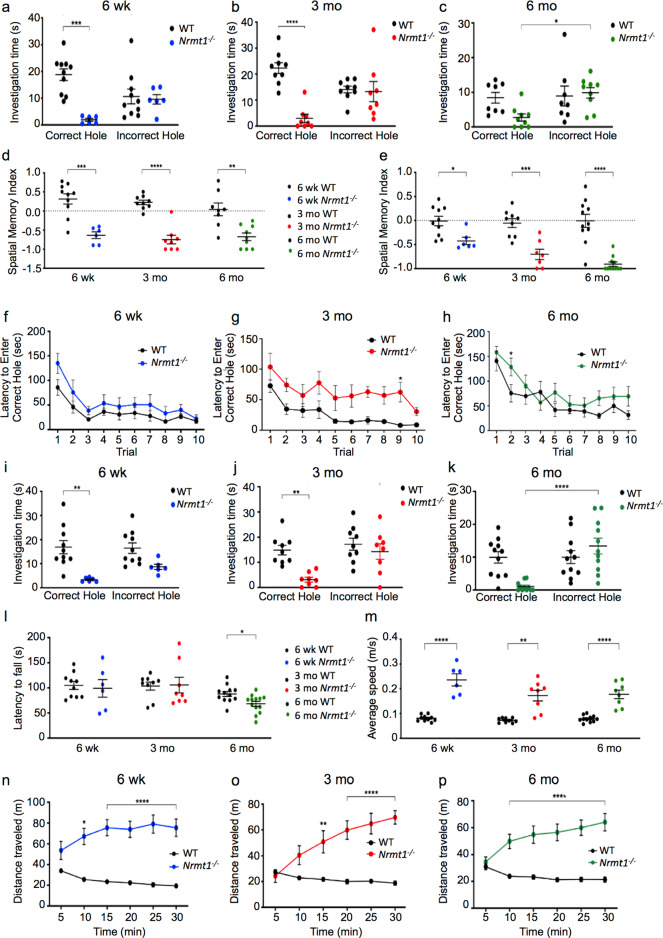
*Nrmt1*^*−/−*^ mice exhibit short- and long-term memory impairment and hyperactivity. Two-trial Barnes maze spatial memory tasks show that *Nrmt1*^−/−^ mice spend significantly less time exploring the correct hole than WT mice at **a** 6 weeks and **b** 3 months. **c** At 6 months, *Nrmt1*^−/−^ mice spend significantly more time at the incorrect holes. **d** The spatial memory indexes (SMI) calculated from the two-trial tasks indicate short-term memory loss at all three ages. SMI = (time exploring correct hole − time exploring incorrect holes)/(total exploration time). **e** The SMIs calculated from the ten-trial tasks also indicate long-term memory loss at all three ages. Although *Nrmt1*^−/−^ mice are able to learn the correct hole when given ten training trials at **f** 6 weeks, **g** 3 months, and **h** 6 months, the ten-trial Barnes maze also shows *Nrmt1*^−/−^ mice spend significantly less time exploring the correct hole at **i** 6 weeks and **j** 3 months, and significantly more time at incorrect holes at **k** 6 months. **l** Rotarod assays show a small motor impairment in *Nrmt1*^−/−^ mice at 6 months. Open-field assays show *Nrmt1*^−/−^ mice travel at **m** higher average speeds for **n**–**p** longer distances than WT mice. *p < 0.05, **p < 0.005, ***p < 0.0005, and ****p < 0.0001 as determined by **a**–**c**, **i**–**k** two-way ANOVA or **d**–**h**, **l**–**p** unpaired *t*-test, *n* = 6–10. Error bars represent mean ± SEM.

Enlargement of the lateral ventricles and/or neurodegeneration of specific brain regions are common pathologies seen in many types of brain disorders including schizophrenia, AD, Parkinson’s disease, and Huntington’s disease [22–25]. Many of these disorders are associated with memory or motor impairments. As the hippocampus contributes to many aspects of short-term, long-term, and spatial memory [26–28], and the striatum is the major input for voluntary motor control [29], we wanted to see whether *Nrmt1*^−/−^ mice similarly exhibited memory or motor impairments. First, we used a two-trial Barnes maze test to assess short-term spatial memory. At 6 weeks and 3 months, *Nrmt1*^−/−^ mice spent significantly less time exploring the correct hole than WT controls during the test trials (Fig. 3a, b). Furthermore, by 6 months, *Nrmt1*^−/−^ mice spent significantly more time at the incorrect holes than the correct hole (Fig. 3c). To summarize the test trial data, spatial memory indices were calculated for all ages. A negative spatial memory index indicates the animal did not remember the escape hatch during the test trial. At all ages, *Nrmt1*^−/−^ mice exhibited negative spatial memory indices that were significantly decreased from WT controls (Fig. 3d), indicating *Nrmt1*^−/−^ mice have short-term spatial memory deficits.

To rule out the possibility that these animals have learning impairments rather than memory deficits, a ten-training trial Barnes maze test was performed and this test also showed significantly decreased spatial memory indices in *Nrmt1*^−/−^ mice at all ages (Fig. 3e). First, mice were given ten 3 min training periods to learn the correct escape hatch from the maze. Although the *Nrmt1*^−/−^ mice progressed somewhat slower, by the tenth trial they showed no significant difference from control animals in their ability to locate the escape hole at any age (Fig. 3f–h), indicating they could learn the task. Twenty-four hours after the last training trial, the escape hatch was removed and exploration time at the correct and incorrect holes was measured. At 6 weeks and 3 months, *Nrmt1*^−/−^ mice spent significantly less time exploring the correct hole than WT controls (Fig. 3i, j), and at 6 months, *Nrmt1*^−/−^ mice spent significantly more time at the incorrect holes than the correct hole (Fig. 3k), indicating *Nrmt1*^−/−^ mice also have impaired long-term memory.

The inability of *Nrmt1*^−/−^ mice to locate and explore the correct hole in the Barnes maze could also result from impaired motor activity. To test motor performance in *Nrmt1*^−/−^ mice, rotarod tests were performed. Mice were placed on an accelerating rotarod for three training trials, followed by two test trials. The amount of time each animal stayed on the rotarod before falling (latency to fall) was measured. There was no significant difference in the latency to fall between *Nrmt1*^−/−^ and WT mice at 6 weeks or 3 months (Fig. 3l). At 6 months, there was a small, yet significant, decrease in motor performance in the *Nrmt1*^−/−^ mice (Fig. 3l). However, this small difference at 6 months on the rotarod cannot account for the impaired performance of *Nrmt1*^−/−^ mice at earlier ages in the Barnes maze. To confirm these results, we also performed open-field assays to test the motor activity of *Nrmt1*^−/−^ mice. Surprisingly, at all ages, *Nrmt1*^−/−^ mice moved at a significantly higher average speed and traveled significantly longer distances than WT controls (Fig. 3m–p), indicating that *Nrmt1*^−/−^ mice do not have any significant motor impairments, although they do exhibit locomotor hyperactivity. This hyperactive phenotype could result from selective death of dopamine D2 receptors in the striatum, which are responsible for inhibition of movement [30, 31].

### Molecular analysis of the NSC niches

**Fig. 4:**
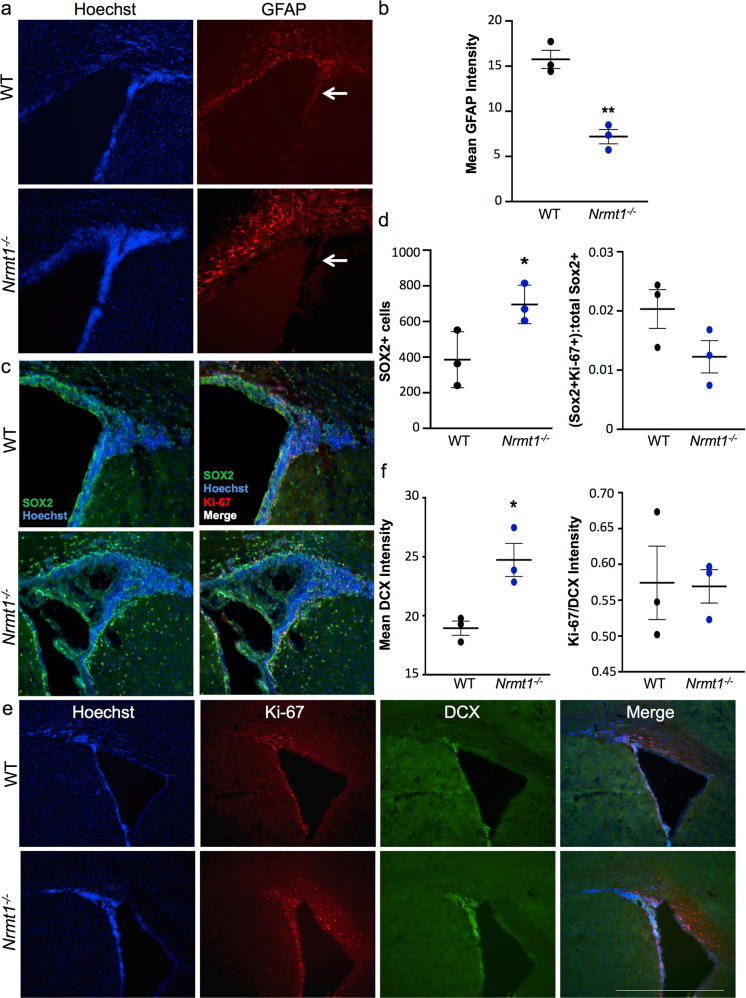
At P14, *Nrmt1*^*−/−*^ mice exhibit an expansion of the IPC and neuroblasts pools in the SVZ. **a**, **b** GFAP staining is significantly decreased in the SVZ (arrows) of *Nrmt1*^*−/−*^ mice. However, there is a significant increase in **c**, **d** SOX2-positive IPCs and **e**, **f** Doublecortin (DCX)-positive neuroblasts in *Nrmt1*^*−/−*^ mice. Neither the **d** percentage of SOX2, Ki-67 double-positive cells, or the **f** ratio of Ki-67/DCX intensity in *Nrmt1*^*−/−*^ mice is significantly different from WT, indicating proliferation is not occurring at these stages. *p < 0.05 and **p < 0.005 as determined by unpaired *t*-test, *n* = 3. Error bars represent mean ± SEM. Scale bar = 1000 μm.

The morphological changes seen in the lateral ventricles and hippocampus, and the corresponding behavioral phenotypes, suggested a potential misregulation of adult NSC neurogenesis, as the two postnatal NSC niches are found in the SVZ of the lateral ventricles and SGZ of the DG [11]. The SVZ contains radial glia-like stem cells (RGLs) that produce both the ependymal cells (E1 cells) that line the ventricle and NSCs [32]. Increased proliferation of RGLs has been shown to sustain ventricle enlargement in mouse model of hydrocephalus [33]. To determine whether there was an expansion of the RGL population in *Nrmt1*^−/−^ mice, we stained ventricular sections of P14 mice with antibodies against GFAP (a marker of both RGLs and astrocytes). Surprisingly, GFAP staining in the SVZ was significantly decreased in *Nrmt1*^−/−^ mice (Fig. 4a, b).

As the RGLs in the *Nrmt1*^−/−^ mice could have prematurely begun differentiation, we also stained ventricle sections of P14 mice with antibodies against SOX2, a marker of both RGLs and IPCs. We found a significant increase in SOX2-positive (SOX2+) cells in and around the SVZ in *Nrmt1*^−/−^ mice as compared to WT (Fig. 4c, d). As GFAP is a marker for RGLs and SOX2 marks both RGLs and IPCs [34], the decrease in GFAP staining and increase in SOX2+ cells in *Nrmt1*^−/−^ mice indicate a shift from normal basal neurogenic activity to an aberrant expansion of IPCs. However, <2% of the SOX2+ cells co-stained with the proliferative marker Ki-67 and this was not significantly different from WT mice, indicating abnormal proliferation is not occurring at the IPC stage (Fig. 4c, d). To determine whether the differentiation of these NSCs is proceeding past the IPC stage to form neuroblasts, we performed immunostaining for the neuroblast marker DCX. There was a significant increase in DCX staining in the ventricle of *Nrmt1*^−/−^ mice (Fig. 4e, f), but the Ki-67/DCX ratio was not significantly different (Fig. 4f), indicating the neuroblast population is also not abnormally proliferative. Taken together, these data indicate expansion of both the IPC and neuroblast pools in the SVZ of *Nrmt1*^−/−^ mice without increased proliferation at these stages. Similar NSC phenotypes were seen in the SGZ (Supplemental Fig. 1).

**Fig. 5:**
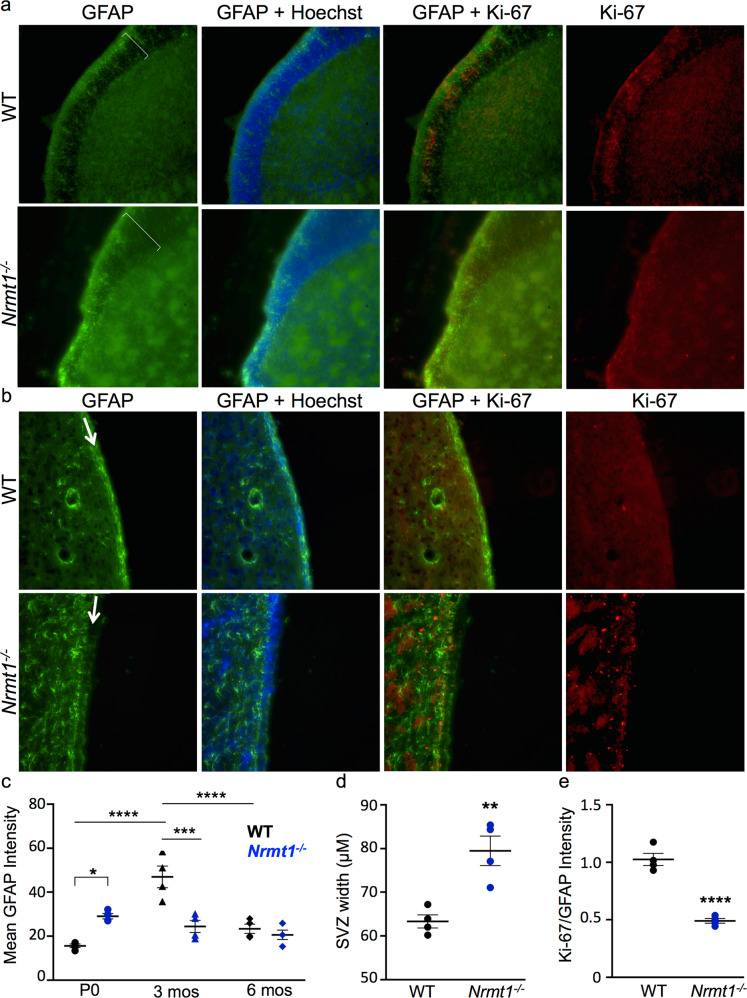
An early expansion of the RGL pool in the SVZ of *Nrmt1*^*−/−*^ mice is followed by premature depletion. **a**, **c** At P0, there is a significant increase in GFAP staining in the SVZ (brackets) of *Nrmt1*^*−/−*^ mice. **b**, **c** At 3 months, GFAP staining is significantly decreased in the SVZ (arrows) of *Nrmt1*^*−/−*^ mice, indicating the early expanded GFAP+ pool has become depleted. **d**, **e** Although the width of the SVZ and intensity of GFAP staining are significantly higher in *Nrmt1*^*−/−*^ mice at P0, the ratio of Ki-67/GFAP is lower than WT, indicating the GFAP+ cells are not undergoing higher rates of proliferation in *Nrmt1*^*−/−*^ mice. **p < 0.005, ***p < 0.0005, and ****p < 0.0001 as determined by unpaired *t*-test, *n* = 3–4. Error bars represent mean ± SEM. Scale bar = 1000 μm.

The lack of increased proliferation in the SOX2+ and DCX+ populations suggests that proliferative expansion of the NSCs came before these stages of differentiation. At P14, we see a decrease in the GFAP staining in the SVZ of *Nrmt1*^−/−^ mice (Fig. 4b), indicating the RGLs have already begun differentiation and an earlier expansion of GFAP+ cells must have occurred. We immunostained brain sections from WT and *Nrmt1*^−/−^ mice from 0-day-old postnatal mice (P0) with GFAP and found a significant increase in GFAP intensity in *Nrmt1*^−/−^ mice (Fig. 5a, c), indicating abnormal expansion of the RGL pool. However, despite the high GFAP staining and increased width of the SVZ (Fig. 5d), the Ki-67/GFAP ratio is significantly lower in *Nrmt1*^−/−^ mice (Fig. 5e), indicating the proliferative expansion of GFAP+ cells must be occurring embryonically.

To see whether the RGL depletion at P14 is maintained as animals age, we also immunostained brain sections from 3- and 6-month-old mice. At 3 months, there is still a significant decrease in GFAP intensity in the SVZ of *Nrmt1*^−/−^ mice (Fig. 5b, c), but by 6 months the GFAP intensity in WT mice decreases to that seen in the knockouts (Fig. 5c and Supplemental Fig. 2), indicating the GFAP+ population normally decreases with age but does so prematurely in *Nrmt1*^−/−^ mice. There is also very little overlap between GFAP and Ki-67 staining in the SVZ of either WT or *Nrmt1*^−/−^ mice at 3 or 6 months, indicating proliferation of this population has mostly ceased by these ages (Fig. 5b and Supplemental Fig. 2). Taken together, these data indicate abnormal embryonic proliferation of the GFAP+ population in *Nrmt1*^−/−^ mice, followed by premature entry into the differentiation pathway before P14. This results in a postnatal expansion of both the IPC and neuroblast pools, and a depletion of the GFAP+ RGL population that persists throughout aging.

### Functional analysis of RB protein in *Nrmt1*^−/−^ mice

**Fig. 6:**
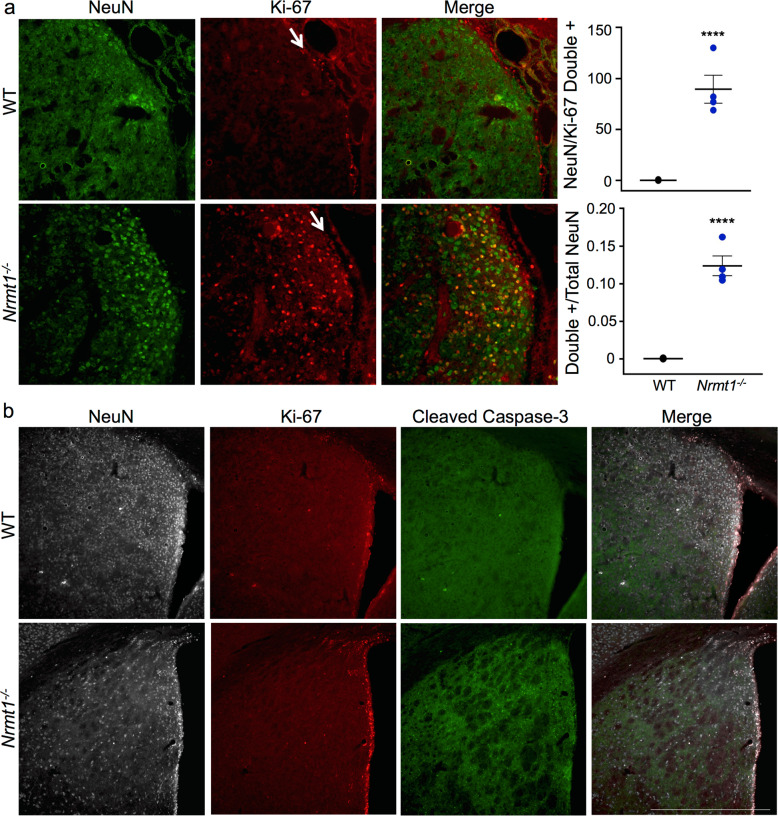
Neurons in *Nrmt1*^*−/−*^ mice remain in the cell cycle and ultimately undergo apoptosis. **a** At P14, Ki-67 staining (red) is restricted to the SVZ (arrow) in WT mice. In *Nrmt1*^*−/−*^ mice, Ki-67+ cells are also found in the neighboring striatum and significantly more of these Ki-67+ cells co-stain (yellow) with NeuN (green), a marker of mature neurons. Over 10% of NeuN-positive cells co-stain with Ki-67. **b** Punctate cleaved caspase-3 staining (green) appears in *Nrmt1*^*−/−*^ mice by 6 weeks and this coincides with the loss of NeuN (gray) and Ki-67 (red) double-positive neurons (pink) in the striatum. ****p < 0.0001 as determined by unpaired *t*-test, *n* = 4. Error bars represent mean ± SEM. Scale bar = 1000 μm.

The expansion of IPC and neuroblast populations seen in *Nrmt1*^−/−^ mice was extremely reminiscent of phenotypes seen in mice deficient for the NRMT1 target protein RB [12–16]. Also seen with RB loss in neuronal lineages is abnormal cell cycle regulation and apoptosis [13, 15]. To determine whether neurons in *Nrmt1*^−/−^ mice exhibited abnormal cell cycle activity, we co-stained ventricle/striatal sections from P14 mice with NeuN (a marker of mature neurons) and Ki-67. At P14, Ki-67 staining was restricted to the SVZ in WT mice and no mature neurons in the striatum co-stained with NeuN and Ki-67 (Fig. 6a). However, in *Nrmt1*^−/−^ mice, Ki-67+ cells were also present in the striatum and there were significantly more NeuN/Ki-67 double-positive cells (Fig. 6a). In total, over 10% of NeuN+ cells co-stained with Ki-67 in *Nrmt1*^−/−^ mice (Fig. 6a). These data indicate loss of NRMT1, similar to loss of RB, results in mature neurons that have abnormal cell cycle regulation. To see whether there is also increased apoptosis in the striatum of *Nrmt1*^−/−^ mice, we stained ventricle/striatal sections for the apoptotic marker cleaved caspase-3. Very little cleaved caspase-3 staining is visible at P14, 3 weeks, or 4 weeks in WT or *Nrmt1*^−/−^ mice (data not shown). However, at 6 weeks, although cleaved caspase-3 staining remains low in WT mice, it increases in the *Nrmt1*^−/−^ mice and this increase corresponds to a decrease in Ki-67/NeuN double-positive cells in the striatum (Fig. 6b). These data indicate that, as with RB deficiency, neurons in *Nrmt1*^−/−^ mice abnormally remain in the cell cycle and ultimately undergo a delayed apoptosis.

**Fig. 7:**
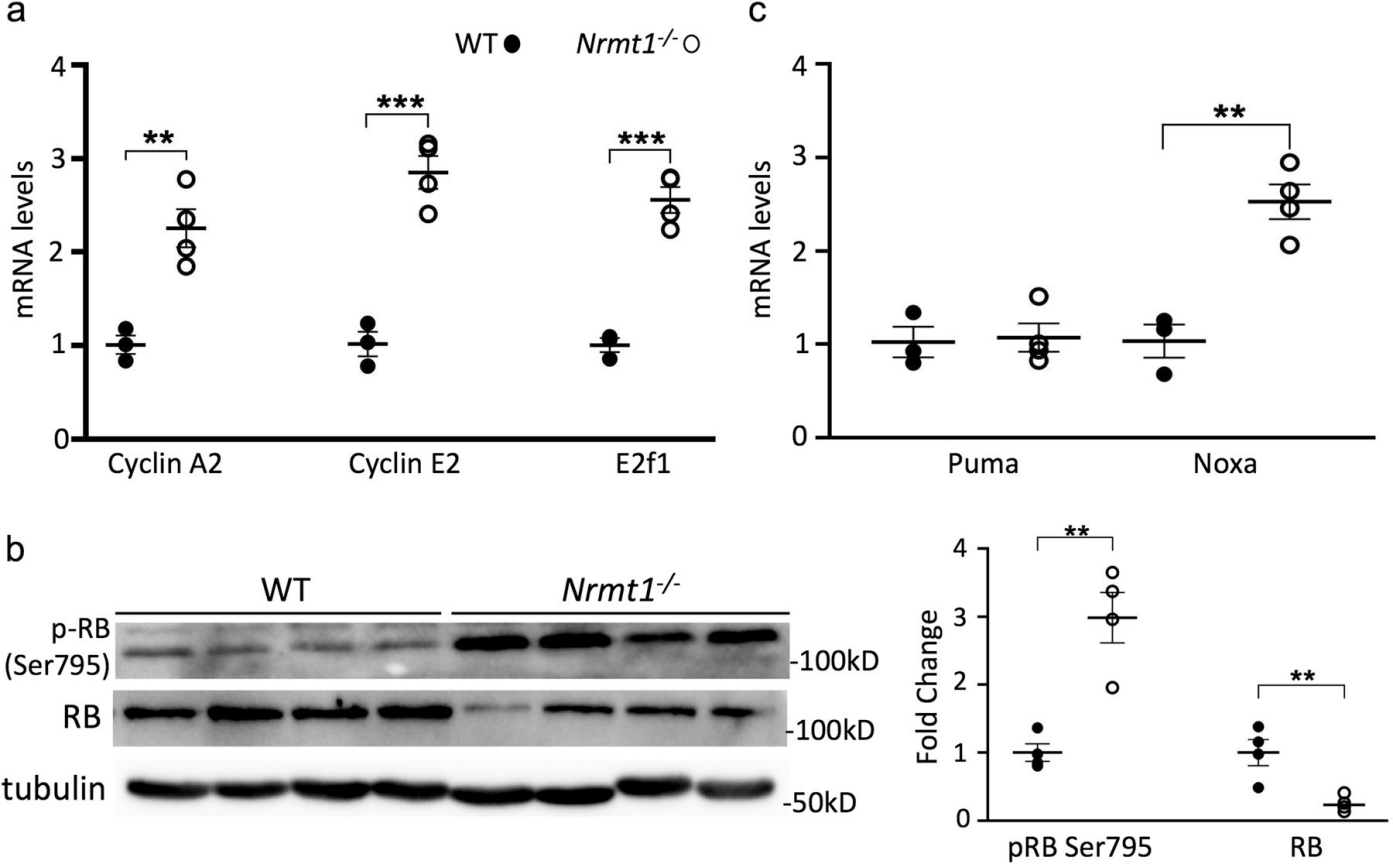
*Nrmt1*^*−/−*^ mice show misregulation of RB function. **a** mRNA levels of RB target genes cyclin A2, cyclin E2, and *E2f1* are significantly increased in *Nrmt1*^*−/−*^ mice. **b** In addition, RB phosphorylation at serine 795 (p-RB Ser795) significantly increases but overall RB protein levels significantly decrease. Quantification of Western blots shown at the right. All samples normalized to tubulin loading control. **c** qRT-PCR analysis indicates apoptosis is not driven through increased expression of the RB target *Puma* but instead by the RB target *Noxa*. **p < 0.005 and ***p < 0.0005 as determined by unpaired *t*-test, *n* = 3–4. Error bars represent mean ± SEM.

RB normally acts to inhibit the cell cycle through its repression of E2F-mediated transcription of cell cycle regulatory genes, including many cyclins, cell division genes, and even *E2f1* itself [35–37]. To monitor whether RB function is disrupted in *Nrmt1*^−/−^ mice, we looked at mRNA expression of cyclin A2, cyclin E2, and *E2f1*. Consistent with expression patterns found with RB deficiency [13, 38], we found that expression of all three cell cycle genes is increased in *Nrmt1*^−/−^ mice (Fig. 7a). We then used Western blots to determine whether this transcriptional de-repression of RB target genes corresponded to changes in RB phosphorylation or protein levels. In *Nrmt1*^−/−^ mice, phosphorylation of RB at Ser795 significantly increases, whereas overall RB protein levels significantly decrease (Fig. 7b), indicating that loss of Nα-methylation triggers the release of RB from E2F proteins and its subsequent degradation [14, 35].

It has been hypothesized that the neuronal apoptosis seen with RB loss is triggered by transcriptional de-repression of the RB/E2F target genes *Puma* and *Apaf1*, but increased expression of these genes was not detected in RB-deficient neurons grown in culture [13]. Similarly, there was no detectable difference in *Puma* levels between WT and *Nrmt1*^−/−^ ventricle/striatal samples (Fig. 7c), and *Apaf1* transcripts were not detectable in either genotype (data not shown). However, RB/E2F also transcriptionally regulate expression of the apoptotic driver *Noxa* [39]. Although p53 upregulated modulator of apoptosis (PUMA) is a direct activator of BAX-induced apoptosis and NOXA is an inhibitor of the anti-apoptotic protein MCL1 [40], they have both been shown to differentially participate in dual pathways of p53-mediated apoptosis induction [41]. Interestingly, we do see a significant increase in *Noxa* expression in *Nrmt1*^−/−^ mice (Fig. 7c), indicating PUMA and NOXA may also play differential roles in RB-mediated apoptosis induction. Taken together, our data suggest that misregulation of RB-dependent cell cycle arrest in NSCs contributes to the morphological and behavior phenotypes seen in *Nrmt1*^−/−^ mice.

## Discussion

Postnatal neurogenesis promotes plasticity and provides a source for replacement of cells lost to turnover or injury [42–44]. Here we show for the first time that loss of NRMT1 results in early expansion and differentiation of quiescent NSCs, which ultimately depletes the postnatal NSC pool. This is followed by morphological and behavioral abnormalities, and ultimately striatal and hippocampal neurodegeneration. We propose that misregulation of RB in *Nrmt1*^−/−^ mice contributes to these phenotypes by promoting both abnormal entry of quiescent NSCs into the cell cycle and apoptosis of terminally differentiated neurons that cannot completely withdraw from the cell cycle. We also propose that other brain regions, such as the cortex, are less affected by loss of NRMT1, because they are less reliant on NSCs from the SVZ or SGZ for postnatal neurogenesis [45, 46].

NRMT1 (also known as METTL11A) is a member of the Methyltransferase like (METTL) family of methyltransferases, and although we show the first identified role for NRMT1 in NSC fate determination, there is an abundance of recent data showing roles for different members of the METTL family in stem cell development. The *N*^6^-methyladenosine (m^6^A) methyltransferases METTL3 and METTL14 regulate the self-renewal of adult hematopoietic stem cells by promoting the expression of genes that maintain quiescence [47]. The more recently discovered m^6^A methyltransferase METTL5 is needed for embryonic stem cell pluripotency [48]. Its loss results in decreased global translation rates and lowered differentiation potential [48]. METTL17, a mitochondrial RNA methyltransferase, regulates the stability of 12S mt-rRNA and its associated proteins, and is required for differentiation of mouse embryonic stem cells [49]. The closest sequence homolog to NRMT1, NRMT2 (also known as METTL11B), is an Nα-monomethylase, and its transcript levels have been shown to increase during osteocytic and myogenic differentiation [50]. We have seen a similar upregulation of NRMT1 transcript levels during myogenic differentiation [51].

Similar to other METTL family members, our data indicate that NRMT1 normally acts to constrain cell cycle activity. We propose it does this through its activation of RB transcriptional repression. As Nα-methylation regulates DNA/protein interactions, our model posits that loss of NRMT1 disrupts the interaction of the N-terminal RB tail with DNA at target promoters and alters the conformation of the RB N-terminal domain (RbN). When RbN interacts with the phosphorylated RB interdomain linker (RbIDL), they are able to bind the RB pocket domain and displace E2F [52]. We propose loss of Nα-methylation of the RB tail promotes interaction between RbN and RbIDL, and displacement of RB from E2F. This leads to increased phosphorylation and degradation of RB and subsequent activation of E2F target genes that promote cell cycle.

We have shown the first role for NRMT1 in neural development, neurodegeneration, and NSC regulation. However, the phenotypes described here result from constitutive NRMT1 loss. It will be interesting to see whether acute NRMT1 loss and subsequent restoration, respectively, stimulate NSC proliferation and allow withdrawal of neurons from the cell cycle. During both neurodegenerative diseases and stroke, the burden of neuronal loss is high and an exhausted or limited pool of stem cells is unable to effectively replace the lost neurons [53]. Understanding whether short-term NRMT1 inhibition could stimulate NSC proliferation and replacement of damaged neurons in these disease states could lead to a novel way of harnessing the potential of endogenous NSCs as therapeutic agents [54, 55].

## Supplementary information

Supplemental Figure 1

Supplemental Figure 1 Legend

Supplemental Figure 2

## Data Availability

The authors confirm that the data supporting the findings of this study are available within the article and its supplementary materials.

## References

[1] Chen T, Muratore TL, Schaner-Tooley CE, Shabanowitz J, Hunt DF, Macara IG (2007). N-terminal alpha-methylation of RCC1 is necessary for stable chromatin association and normal mitosis. Nat Cell Biol.

[2] Cai Q, Fu L, Wang Z, Gan N, Dai X, Wang Y (2014). α-N-methylation of damaged DNA-binding protein 2 (DDB2) and its function in nucleotide excision repair. J Biol Chem.

[3] Bailey AO, Panchenko T, Sathyan KM, Petkowski JJ, Pai PJ, Bai DL (2013). Posttranslational modification of CENP-A influences the conformation of centromeric chromatin. Proc Natl Acad Sci USA.

[4] Tooley CE, Petkowski JJ, Muratore-Schroeder TL, Balsbaugh JL, Shabanowitz J, Sabat M (2010). NRMT is an alpha-N-methyltransferase that methylates RCC1 and retinoblastoma protein. Nature.

[5] Petkowski JJ, Schaner Tooley CE, Anderson LC, Shumilin IA, Balsbaugh JL, Shabanowitz J (2012). Substrate specificity of mammalian N-terminal alpha-amino methyltransferase NRMT. Biochemistry.

[6] Cai Q, Fu L, Wang Z, Gan N, Dai X, Wang Y (2014). alpha-N-methylation of damaged DNA-binding protein 2 (DDB2) and its function in nucleotide excision repair. J Biol Chem.

[7] Dai X, Otake K, You C, Cai Q, Wang Z, Masumoto H (2013). Identification of novel alpha-n-methylation of CENP-B that regulates its binding to the centromeric DNA. J Proteome Res.

[8] Bonsignore LA, Butler JS, Klinge CM, Schaner Tooley CE (2015). Loss of the N-terminal methyltransferase NRMT1 increases sensitivity to DNA damage and promotes mammary oncogenesis. Oncotarget.

[9] Bonsignore LA, Tooley JG, Van Hoose PM, Wang E, Cheng A, Cole MP (2015). NRMT1 knockout mice exhibit phenotypes associated with impaired DNA repair and premature aging. Mech Ageing Dev.

[10] Coppede F, Migliore L (2010). DNA repair in premature aging disorders and neurodegeneration. Curr Aging Sci.

[11] Obernier K, Alvarez-Buylla A. Neural stem cells: origin, heterogeneity and regulation in the adult mammalian brain. *Development*. 2019;146:dev156059. 10.1242/dev.156059 PMC6398449 30777863

[12] Ferguson KL, Vanderluit JL, Hébert JM, McIntosh WC, Tibbo E, MacLaurin JG (2002). Telencephalon-specific Rb knockouts reveal enhanced neurogenesis, survival and abnormal cortical development. EMBO J.

[13] Andrusiak MG, Vandenbosch R, Park DS, Slack RS (2012). The retinoblastoma protein is essential for survival of postmitotic neurons. J Neurosci.

[14] Park DS, Morris EJ, Bremner R, Keramaris E, Padmanabhan J, Rosenbaum M (2000). Involvement of retinoblastoma family members and E2F/DP complexes in the death of neurons evoked by DNA damage. J Neurosci.

[15] Naser R, Vandenbosch R, Omais S, Hayek D, Jaafar C, Al Lafi S (2016). Role of the retinoblastoma protein, Rb, during adult neurogenesis in the olfactory bulb. Sci Rep.

[16] Vandenbosch R, Clark A, Fong BC, Omais S, Jaafar C, Dugal-Tessier D (2016). RB regulates the production and the survival of newborn neurons in the embryonic and adult dentate gyrus. Hippocampus.

[17] Ploner C, Kofler R, Villunger A (2008). Noxa: at the tip of the balance between life and death. Oncogene.

[18] Pompl PN, Mullan MJ, Bjugstad K, Arendash GW (1999). Adaptation of the circular platform spatial memory task for mice: use in detecting cognitive impairment in the APP(SW) transgenic mouse model for Alzheimer’s disease. J Neurosci Methods.

[19] Dunham NW, Miya TS (1957). A note on a simple apparatus for detecting neurological deficit in rats and mice. J Am Pharm Assoc Am Pharm Assoc.

[20] Madabhushi R, Pan L, Tsai LH (2014). DNA damage and its links to neurodegeneration. Neuron.

[21] Horga G, Bernacer J, Dusi N, Entis J, Chu K, Hazlett EA (2011). Correlations between ventricular enlargement and gray and white matter volumes of cortex, thalamus, striatum, and internal capsule in schizophrenia. Eur Arch psychiatry Clin Neurosci.

[22] Wright IC, Rabe-Hesketh S, Woodruff PW, David AS, Murray RM, Bullmore ET (2000). Meta-analysis of regional brain volumes in schizophrenia. Am J Psychiatry.

[23] Guptha SH, Holroyd E, Campbell G (2002). Progressive lateral ventricular enlargement as a clue to Alzheimer’s disease. Lancet.

[24] Apostolova L, Alves G, Hwang KS, Babakchanian S, Bronnick KS, Larsen JP (2012). Hippocampal and ventricular changes in Parkinson’s disease mild cognitive impairment. Neurobiol Aging.

[25] Hobbs NZ, Barnes J, Frost C, Henley SM, Wild EJ, Macdonald K (2010). Onset and progression of pathologic atrophy in Huntington disease: a longitudinal MR imaging study. AJNR Am J Neuroradiol.

[26] Kumaran D (2008). Short-term memory and the human hippocampus. J Neurosci.

[27] Squire LR, Zola-Morgan S (1991). The medial temporal lobe memory system. Science.

[28] Burgess N (2002). The hippocampus, space, and viewpoints in episodic memory. Q J Exp Psychol A.

[29] Hikosaka O, Takikawa Y, Kawagoe R (2000). Role of the basal ganglia in the control of purposive saccadic eye movements. Physiol Rev.

[30] Graybiel AM (1990). Neurotransmitters and neuromodulators in the basal ganglia. Trends Neurosci.

[31] DeLong MR (1990). Primate models of movement disorders of basal ganglia origin. Trends Neurosci.

[32] Redmond SA, Figueres-Oñate M, Obernier K, Nascimento MA, Parraguez JI, López-Mascaraque L (2019). Development of ependymal and postnatal neural stem cells and their origin from a common embryonic progenitor. Cell Rep.

[33] Bátiz LF, Jiménez AJ, Guerra M, Rodríguez-Pérez LM, Toledo CD, Vio K (2011). New ependymal cells are born postnatally in two discrete regions of the mouse brain and support ventricular enlargement in hydrocephalus. Acta Neuropathol.

[34] Hutton SR, Pevny LH (2011). SOX2 expression levels distinguish between neural progenitor populations of the developing dorsal telencephalon. Dev Biol.

[35] DeGregori J (2002). The genetics of the E2F family of transcription factors: shared functions and unique roles. Biochim Biophys Acta.

[36] White J, Stead E, Faast R, Conn S, Cartwright P, Dalton S (2005). Developmental activation of the Rb-E2F pathway and establishment of cell cycle-regulated cyclin-dependent kinase activity during embryonic stem cell differentiation. Mol Biol Cell.

[37] Neuman E, Flemington EK, Sellers WR, Kaelin WG (1995). Transcription of the E2F-1 gene is rendered cell cycle dependent by E2F DNA-binding sites within its promoter. Mol Cell Biol.

[38] Ghanem N, Andrusiak MG, Svoboda D, Al Lafi SM, Julian LM, McClellan KA (2012). The Rb/E2F pathway modulates neurogenesis through direct regulation of the Dlx1/Dlx2 bigene cluster. J Neurosci.

[39] Hershko T, Ginsberg D (2004). Up-regulation of Bcl-2 homology 3 (BH3)-only proteins by E2F1 mediates apoptosis. J Biol Chem.

[40] Hollville E, Romero SE, Deshmukh M (2019). Apoptotic cell death regulation in neurons. FEBS J.

[41] Shibue T, Suzuki S, Okamoto H, Yoshida H, Ohba Y, Takaoka A (2006). Differential contribution of Puma and Noxa in dual regulation of p53-mediated apoptotic pathways. EMBO J.

[42] Ma DK, Bonaguidi MA, Ming GL, Song H (2009). Adult neural stem cells in the mammalian central nervous system. Cell Res.

[43] Nissant A, Bardy C, Katagiri H, Murray K, Lledo PM (2009). Adult neurogenesis promotes synaptic plasticity in the olfactory bulb. Nat Neurosci.

[44] Kitabatake Y, Sailor KA, Ming GL, Song H (2007). Adult neurogenesis and hippocampal memory function: new cells, more plasticity, new memories?. Neurosurg Clin North Am.

[45] De Marchis S, Fasolo A, Puche AC (2004). Subventricular zone-derived neuronal progenitors migrate into the subcortical forebrain of postnatal mice. J Comp Neurol.

[46] Dayer AG, Cleaver KM, Abouantoun T, Cameron HA (2005). New GABAergic interneurons in the adult neocortex and striatum are generated from different precursors. J Cell Biol.

[47] Yao QJ, Sang L, Lin M, Yin X, Dong W, Gong Y (2018). Mettl3-Mettl14 methyltransferase complex regulates the quiescence of adult hematopoietic stem cells. Cell Res.

[48] Ignatova VV, Stolz P, Kaiser S, Gustafsson TH, Lastres PR, Sanz-Moreno A (2020). The rRNA m(6)A methyltransferase METTL5 is involved in pluripotency and developmental programs. Genes Dev.

[49] Shi Z, Xu S, Xing S, Yao K, Zhang L, Xue L (2019). Mettl17, a regulator of mitochondrial ribosomal RNA modifications, is required for the translation of mitochondrial coding genes. FASEB J.

[50] Hong AR, Kim K, Lee JY, Yang JY, Kim JH, Shin CS (2020). Transformation of mature osteoblasts into bone lining cells and RNA sequencing-based transcriptome profiling of mouse bone during mechanical unloading. Endocrinol Metab.

[51] Tooley JG, Catlin JP, Tooley CES. CREB-mediated transcriptional activation of NRMT1 drives muscle differentiation. Transcription. 2021;1–17. 10.1080/21541264.2021.1963627. 10.1080/21541264.2021.1963627 PMC8555533 34403304

[52] Burke JR, Deshong AJ, Pelton JG, Rubin SM (2010). Phosphorylation-induced conformational changes in the retinoblastoma protein inhibit E2F transactivation domain binding. J Biol Chem.

[53] Holvoet B, De Waele L, Quattrocelli M, Gheysens O, Sampaolesi M, Verfaillie CM (2016). Increased understanding of stem cell behavior in neurodegenerative and neuromuscular disorders by use of noninvasive cell imaging. Stem Cells Int.

[54] Sugaya K, Vaidya M (2018). Stem cell therapies for neurodegenerative diseases. Adv Exp Med Biol.

[55] Huang L, Zhang L (2019). Neural stem cell therapies and hypoxic-ischemic brain injury. Prog Neurobiol.

