# Supplementary figures and images for: Age-related neurodegeneration and cognitive impairments of NRMT1 knockout mice are preceded by misregulation of RB and abnormal neural stem cell development
Source: Cell Death Dis. 2021 Oct 28;12(11):1014. doi: 10.1038/s41419-021-04316-0 (PMC8553844; doi:10.1038/s41419-021-04316-0)

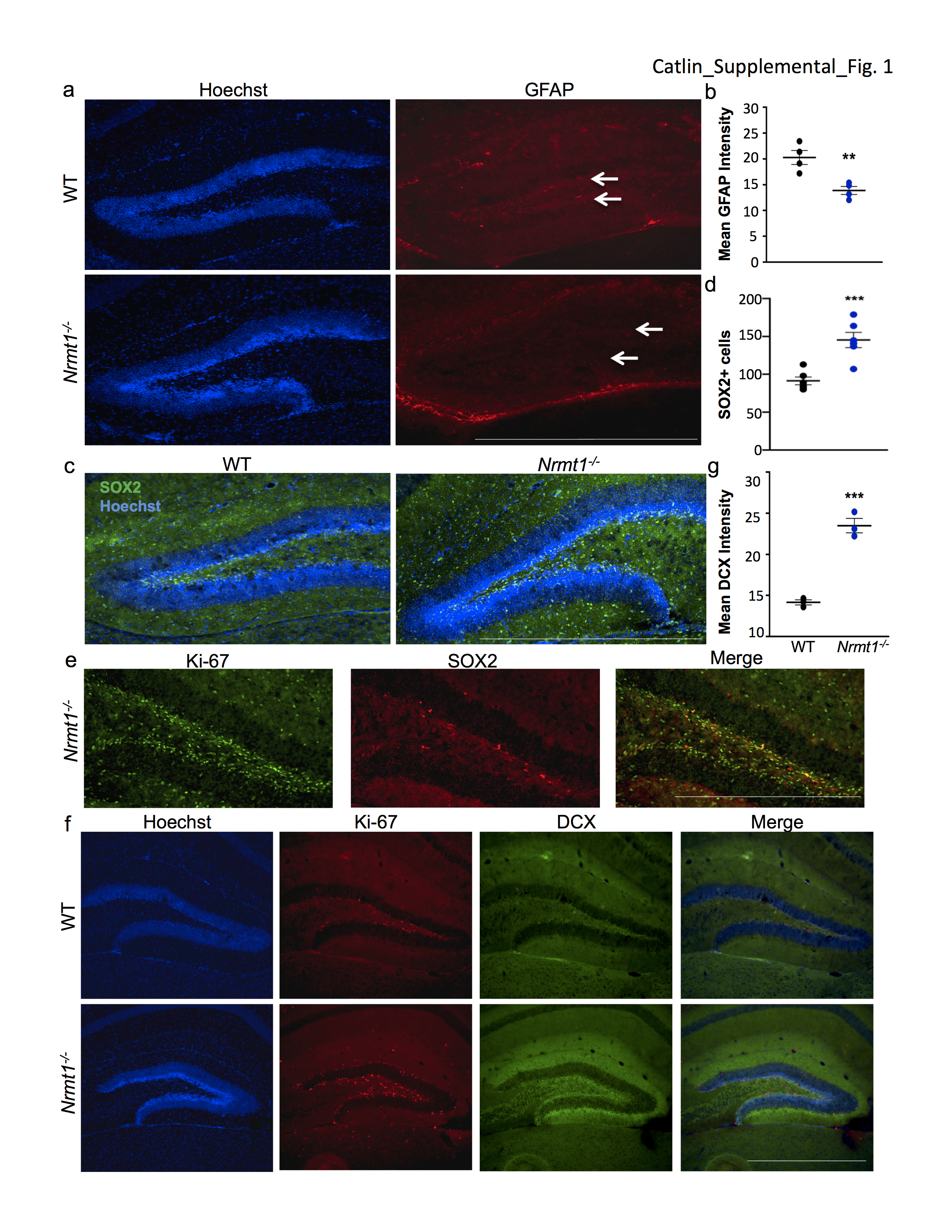

Supplement: Supplementary file 1 — Supplemental Figure 1 [file 41419_2021_4316_MOESM1_ESM.png]

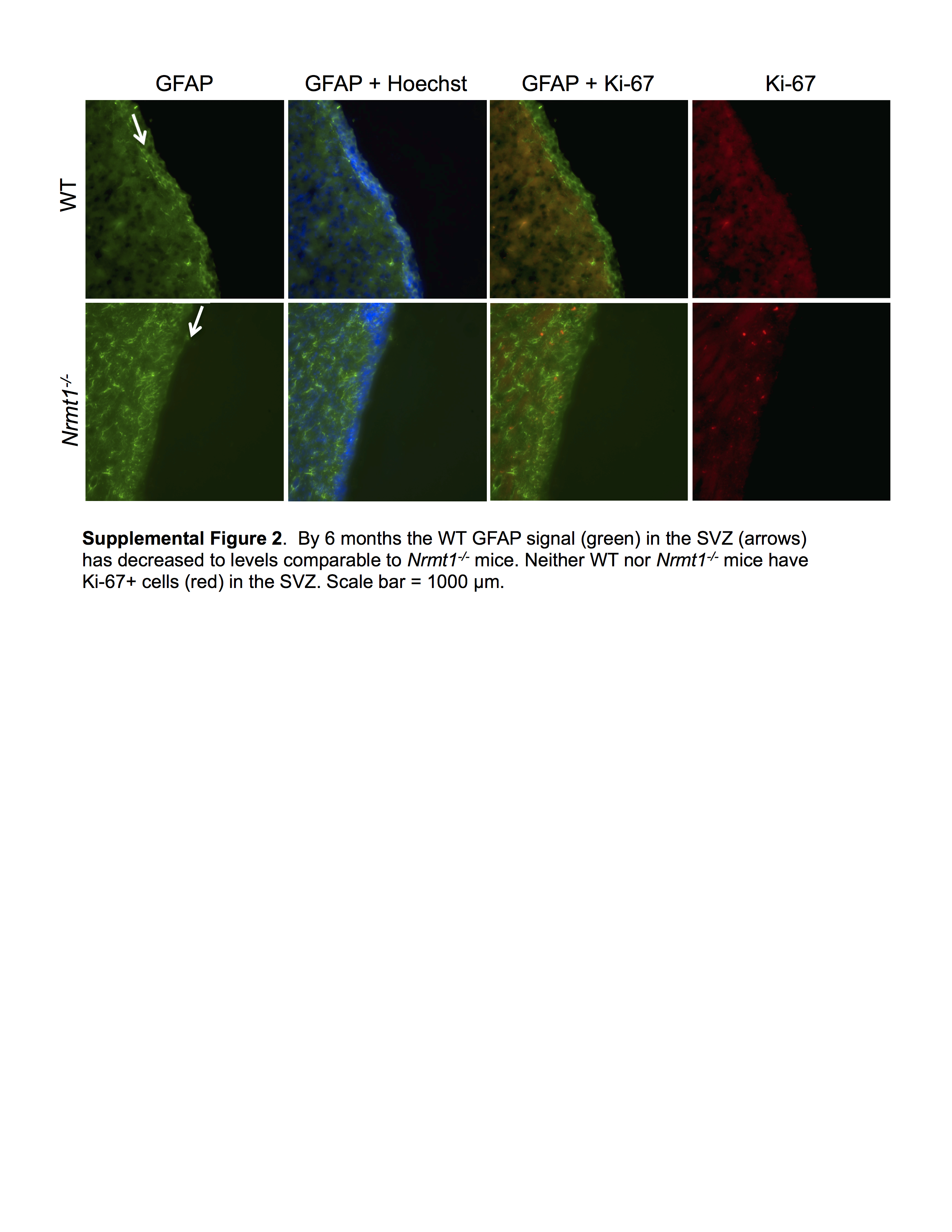

Supplement: Supplementary file 3 — Supplemental Figure 2 [file 41419_2021_4316_MOESM3_ESM.png]
