# Supplementary material for: Age-related neurodegeneration and cognitive impairments of NRMT1 knockout mice are preceded by misregulation of RB and abnormal neural stem cell development
Source: Cell Death Dis. 2021 Oct 28;12(11):1014. doi: 10.1038/s41419-021-04316-0 (PMC8553844; doi:10.1038/s41419-021-04316-0)
Supplement: Supplementary file 2 — Supplemental Figure 1 Legend [file 41419_2021_4316_MOESM2_ESM.pdf]

Supplemental Figure 1. **a,b** As seen in the SVZ, there is a significant decrease in GFAP intensity in the SGZ of *Nrmt1*$^{-/-}$ mice. **c,d** SOX2+ cells are also significantly increased in the SGZ of *Nrmt1*$^{-/-}$ mice, (**e**) but there is little overlap between the SOX2 and Ki-67 staining patterns. **f,g** DCX immunostaining is also increased. ** denotes $p<0.005$ and *** denotes $p<0.0005$ as determined by unpaired t-test, n=4-6. Error bars represent mean ± SEM. Scale bar = 1000 μm.
